# Protection of Rabbits and Immunodeficient Mice against Lethal Poxvirus Infections by Human Monoclonal Antibodies

**DOI:** 10.1371/journal.pone.0048706

**Published:** 2012-11-02

**Authors:** Lindsay Crickard, Tahar Babas, Sidharth Seth, Peter Silvera, Lilia Koriazova, Shane Crotty

**Affiliations:** 1 Division of Vaccine Discovery, La Jolla Institute for Allergy and Immunology (LIAI), La Jolla, California, United States of America; 2 Infectious Disease Research Department, Southern Research Institute, Frederick, Maryland, United States of America; 3 Kyowa Hakko Kirin California, Inc., La Jolla, California, United States of America; 4 Department of Medicine, University of California San Diego School of Medicine, La Jolla, California, United States of America; The University of Hong Kong, Hong Kong

## Abstract

Smallpox (variola virus) is a bioweapon concern. Monkeypox is a growing zoonotic poxvirus threat. These problems have resulted in extensive efforts to develop potential therapeutics that can prevent or treat potentially lethal poxvirus infections in humans. Monoclonal antibodies (mAbs) against smallpox are a conservative approach to this problem, as the licensed human smallpox vaccine (vaccinia virus, VACV) primarily works on the basis of protective antibody responses against smallpox. Fully human mAbs (hmAbs) against vaccinia H3 (H3L) and B5 (B5R), targeting both the mature virion (MV) and extracellular enveloped virion (EV) forms, have been developed as potential therapeutics for use in humans. Post-exposure prophylaxis was assessed in both murine and rabbit animal models. Therapeutic efficacy of the mAbs was assessed in three good laboratory practices (GLP) studies examining severe combined immunodeficiency mice (SCID) given a lethal VACV infection. Pre-exposure combination hmAb therapy provided significantly better protection against disease and death than either single hmAb or vaccinia immune globulin (VIG). Post-exposure combination mAb therapy provided significant protection against disease and death, and appeared to fully cure the VACV infection in ≥50% of SCID mice. Therapeutic efficacy was then assessed in two rabbit studies examining post-exposure hmAb prophylaxis against rabbitpox (RPXV). In the first study, rabbits were infected with RPVX and then provided hmAbs at 48 hrs post-infection, or 1 hr and 72 hrs post-infection. Rabbits in both groups receiving hmAbs were 100% protected from death. In the second rabbitpox study, 100% of animal treated with combination hmAb therapy and 100% of animals treated with anti-B5 hmAb were protected. These findings suggest that combination hmAb treatment may be effective at controlling smallpox disease in immunocompetent or immunodeficient humans.

## Introduction

Smallpox is a highly lethal viral infection affecting humans (30% mortality) [1] which can spread rapidly through a population. Smallpox is a top bioterrorism concern and is frequently considered the greatest bioterrorism danger [2], [3]. The smallpox vaccine consists of live vaccinia virus (VACV) and, from a public health perspective, is the gold standard of vaccines because it has led to the complete eradication of wild smallpox (variola virus) from the human population [4]. Renewed fears that smallpox might be deliberately released in an act of bioterrorism have led to resurgence in the study of treatment of smallpox infection. Individuals <35 years old (approximately 50% of the population) have not been vaccinated against smallpox, leaving them highly susceptible in the event of an outbreak. There is also substantial interest in better therapeutics for the treatment of the rare but severe side effects of the smallpox vaccine. Finally, there is also interest in therapeutics for treatment of other poxviruses, such as monkeypox, which is transmitted among rodent populations. A monkeypox outbreak occurred for the first time in the USA in 2003 [5], [6], [7], [8].

The smallpox vaccine is administered as a series of 3–15 skin pricks using a bifurcated needle [3]. Four major smallpox vaccine strains were used during the massive WHO vaccination programme (VACV_NYCBOH_ [USA], Lister [UK], Temple of Heaven [China] and EM-63 [USSR]). In the USA, the vaccine was commercially produced as Dryvax® (also known as the VACV Wyeth strain or substrain). A clonal isolate of VACV_NYCBOH_, ACAM2000®, has now been developed as a cell-culture derived smallpox vaccine, with a comparable immunogenicity and safety profile to Dryvax® [9], [10], and ACAM2000® is now the currently licensed smallpox vaccine in the USA. The vaccine “take” is observed as the formation of a pustule starting on approximately day 5 post-vaccination and lasting for 1–2 weeks thereafter [3], [11], [12]. The vaccine provides outstanding immunity, but could cause a variety of side effects that have been reason for concern [2], [13]. Common side effects include fever and satellite pox (additional pustules near the primary pustule, also called mild generalized vaccinia). More severe side effects include progressive vaccinia, generalized vaccinia, encephalitis, vaccinia keratitis, and eczema vaccinatum [11], [13], [14], [15].

Currently, VIG is the only licensed therapeutic to treat the side effects of smallpox vaccination [2], [13]. In addition, VIG has shown efficacy against smallpox itself in clinical trials in the early 1960s. A meta-analysis of the four available controlled studies carried out with VIG indicates that VIG is protective and reduces smallpox cases by approximately 75% [16]. VIG reduced the spread of smallpox outbreaks when administered at the same time as smallpox vaccination to smallpox contacts [16], [17], [18], [19]. In another study, a smallpox outbreak initially killed 3 out of 10 patients. When patient care was expanded to include administration of high-titre smallpox-specific convalescent serum at the first signs of disease, the mortality rate dropped to 0% (out of 250 subsequent infections reported) [20].

There is also compelling animal model data supporting the efficacy of VIG against pathogenic poxvirus infections. Licensed VIG has demonstrated efficacy by *in vitro* neutralization of VACV and *in vivo* treatment of severe combined immunodeficiency (SCID) mice infected with VACV [2], [21], [22], [23], [24], [25]. In rhesus macaque monkeypox studies, it was demonstrated that smallpox vaccine-elicited neutralizing antibodies were necessary for protection [26]. Furthermore, it was shown that neutralizing antibodies were sufficient for protection against a lethal monkeypox challenge, as administration of VIG to unvaccinated macaques prior to monkeypox challenge provided protection [26]. Although animals developed skin lesions (that is, pox) in a dose-dependent manner with an inverse relationship to the amount of VIG administered, they were all fully protected from lethal infection [26].

Unfortunately, VIG is a poorly characterized, variable human product that is of limited potency [2] [16]. Each of these issues is a major problem for biodefense preparedness against a smallpox bioterrorism event. These problems with VIG have led to interest in the development of an alternative high-potency anti-smallpox immunotherapy free of these issues.

Our goal is to develop a highly efficacious and standardized monoclonal antibody (mAb) –based anti-smallpox therapeutic that can be produced in large quantities and stored long-term. Poxviruses (VACV, variola/smallpox, and monkeypox) have two virion forms, intracellular mature virions (MV, IMV) and extracellular enveloped virions (EV, EEV), each with distinct structure and biology. Importantly, the two virion forms do not share any surface proteins; therefore, the virion forms are immunologically distinct and are not neutralized by antibody of a single specificity [27], [28]. Both anti-MV and anti-EV antibodies can be effective at protection in animal models [27], [28]. VIG contains both anti-MV and anti-EV antibodies [25], [29], [30], [31], [32], [33], [34], [35]; therefore, an effective VIG replacement therapeutic product should contain one anti-MV mAb and one anti-EV mAb, each capable of neutralizing their respective virion form.

In past studies we have reported characterization of mAbs against H3 and B5 (MV and EV antigens, respectively), demonstrating their efficacy against VACV in an *in vivo* model of progressive vaccinia, using VIG as the benchmark for efficacy [36]. In the following studies, we show efficacy of the hmAbs in protecting SCID mice against VACV infection both pre-exposure and post-exposure in studies run under GLP regulations (US FDA 21 CFR part 58). Two post-exposure protection studies of New Zealand White rabbits infected with rabbitpox virus (RPXV) were also completed. These studies show a high degree of protection provided by the hmAb combination or anti-B5 hmAb alone.

## Methods

### Ethics Statement

All mouse experiments were conducted in compliance with the LIAI Institutional Animal Care and Use Committee approved animal protocols and Good Laboratory Practice Regulations (U.S. FDA 21 CFR part 58). All rabbit care and procedures were carried in compliance with the *Guide for the Care and Use of Laboratory Animals*, 8th Edition (Institute of Animal Resources, Commission on Life Sciences, National Research Council; National Academy Press; Washington, DC; 2011), and the U.S. Department of Agriculture through the Animal Welfare Act (Public Law 99–198) in a facility fully accredited by the Association for Assessment and Accreditation of Laboratory Animal Care International (AAALAC). The protocol was approved by the SRI Institutional Animal Care and Use Committee.

### Viruses

A laboratory stock of the ACAM2000® clone of VACV_NYCBOH_ was used in the first mouse experiment. The ACAM2000® stock was generated by single-passage amplification in HeLa cells of ACAM2000® vaccinia virus (Acambis, Inc., Cambridge, MA, USA). For the following mouse experiments ACAM2000® from the strategic national stockpile from the CDC was re-constituted with the provided diluent and used for infection.

Rabbitpox virus (RPXV) stock, Utrecht strain (ATCC, VR-157) was established as tissue culture supernatant of African green monkey kidney cell line CV-1 (ATCC, CCL-70) inoculated with RPXV at 0.5 MOI (multiplicity of infection). When cells reached at least 90% CPE, the supernatant was harvested, clarified by centrifugation, aliquoted and stored in −80°C freezer. Subsequently, RPXV stock was titrated *in vitro* by standard plaque assay on Vero E6 cell line (ATCC, CRL-1586).

### Mice and Infections

Female SCID mice on the BALB/c background (CBy.Smn.CB17_PRKdc_^SCID/J^) were purchased from Jackson Laboratories (Sacramento, CA, USA) and were acclimatized for at least 1 week prior to infection. Mice were used in experiments at approximately 8 weeks of age (approximately 15–25 grams). Animal health was assessed prior to infection. For the first study animals were housed 5 per cage, while in the following two studies animals were housed singly in microisolator cages in ventilated racks. All animal experiments were conducted in compliance with the Institutional Animal Care and Use Committee approved animal protocols and Good Laboratory Practice Regulations (U.S. FDA 21 CFR part 58).

Mice received food and acidified water (pH 2.5–3.5) *ad libitum.* The animals’ diet was irradiated PicoLab® Rodent Diet 20 (catalogue number 5053; Lab Diet). There are no known contaminants in the feed or water that would interfere with the results of the studies. Animals were housed in micro-isolator cages in ventilated racks at a minimum of 50 air changes/per room/per hour. The rooms were ventilated with 100% fresh air. A 12-hour light/12-hour dark cycle was maintained, except when room lights were turned on to accommodate husbandry or other study activities. The room was maintained at 72±2°F and 30–70% relative humidity. Animals were observed within their cages once daily throughout the acclimation period. Each animal was observed for changes in general appearance and behavior. Any abnormal observation was reported to the Study Director. Observations for moribundity and mortality were performed daily. If an animal was found moribund or dead by the vivarium staff, notification was sent immediately to the researcher and study director. Study animals will be terminated once they reach 75% of pre-injection body weight. Euthanasia was performed by administration of CO_2_ asphyxiation.

Mice were given 200–400 µL intraperitoneal, retro-orbital, or combinations of the two injections of antibodies at 18–24 hours prior to or after infection. Mice were infected with ACAM2000® intravenously via the tail vein or retro-orbital route in a 200 µL volume. All mice received 5×10^4^ PFU of virus. Immediately, prior to use, virus (from high-titre stocks stored at −80°C ) was diluted into plain Dulbecco’s modified Eagle’s medium.

### Rabbits and Infections

Nine week-old female New Zealand White (NZW) rabbits purchased from Charles River (Location C22, Canada) were used in efficacy studies against RPXV challenge. All animal care and procedures were carried in compliance with the *Guide for the Care and Use of Laboratory Animals*, 8th Edition (Institute of Animal Resources, Commission on Life Sciences, National Research Council; National Academy Press; Washington, DC; 2011), and the U.S. Department of Agriculture through the Animal Welfare Act (Public Law 99–198) in a facility fully accredited by the Association for Assessment and Accreditation of Laboratory Animal Care International (AAALAC). The protocol was initially approved by the Institutional Animal Care and Use Committee. Animals were single-housed in individual stainless steel cages in an environmentally monitored and ventilated room maintained at a temperature of 61–72°F and a relative humidity of 30%-70%. Fluorescent lighting provided illumination approximately 12 hours per day. Rabbits were fed TEKLAD 2031C Global Certified High Fiber Rabbit Diet (Harlan TEKLAD; Madison, WI) during the quarantine and study periods. Drinking water and feed were provided *ad libitum* throughout the study. In the first study, a total of 32 rabbits distributed into 4 groups were challenged with 1×10^5^ PFU of RPXV via intranasal route. Group 1 (n = 10) did not receive treatment and served as negative control. Group 3 (n = 6) received a single intravenous injection of antibody cocktail at 9 mg/kg on day 2 post-challenge. Groups 2 (n = 6) and 4 (n = 10) received two intravenous injections with antibody cocktail at 9 mg/kg and 1.25 mg/kg, respectively, one hour and 72 hours following challenge. In a second study, a total of 14 rabbits distributed into 3 groups were challenged intranasally with 1×10^5^ PFU of RPXV. Group 1 (n = 6) did not receive treatment and served as the study negative control. Group 2 (n = 6) received two intravenous injections of antibody h101 alone at 9 mg/kg, 1 hour and 72 hours following challenge. Groups 3 (n = 2) received two intravenous injections with the antibody cocktail at 9 mg/kg, 1 hour and 72 hours post-challenge. Following challenge, all animals were monitored daily for clinical signs of disease, body weight and temperature. The criteria for euthanasia included, but were not limited to, open mouth breathing, severe lung sounds, weight loss greater than 15% and hypothermia.

### Mouse Observations

Body Weight. After infecting animals on day 0 or day 1, weights were taken for the initial body weight measurement. Body weights were then taken every other day or once a week, beginning day 3 following dosing. Body weights were recorded until the animal reached 75% of initial body weight in all experiments (experimental end-point) or if other external health variables became present for which euthanasia was the only humane course of action. Studies were ended on day 90 unless otherwise amended.

Clinical Score. Mice developed lesions (pox) on the tail at 6–8 days post-infection. The method of clinical scoring evaluates the number and severity of the lesions [15]. The tail was scored in three parts: the base, middle and tip of the tail. The lesions were evaluated as follows: score 0, no pox; score 1, 1–3 pox; score 2, ≥3 pox and score 3, ≥3 pox and erupted. An additional evaluation of the paws was taken. A score of 1 was given if the animal only had pox on its hind paws and a score of 2 was given when the animal had pox on the front and hind paws. The maximum score was 11 (for example, base = 3; mid = 3; tip = 3 and paws = 2). Clinical scores usually stabilized at 15–20 days post-infection.

### Rabbit Observations

After challenge, all animals in Groups 1–4 were monitored daily for clinical signs of disease for 14 days in the first study, and 12 in the second study. Body weight, temperature, and respiration rate were recorded daily. For body weight collection, animals were weighed every day for the duration of the study. For body temperature collection, a sterile programmable transponder was implanted subcutaneously at the base of the neck of each animal on the day of receipt. The transponder was scanned daily to collect body temperature for the duration of the study. All rabbits were observed daily for changes in behavior or clinical signs of pox disease including nasal and ocular discharges, pox lesions in the ears, nose, mouth, eye lids, and ano-genital area. A numerical score was assigned for each of the clinical observation parameters including weakness, depression, dehydration, dyspnea, edema, nasal and ocular discharges, based on the severity of manifestation. The scoring system scale is 0 = when clinical sign not present, 1 = mild, 2 = moderate and 3 = severe clinical parameter manifestation. Viral load in blood was determined every 2 days for all animals. Animals that exhibited clinical signs of RPXV disease were euthanized if they met specific pre-defined criteria. These criteria included (but were not limited to) open mouth breathing, severe lung sounds, weight loss greater than 15%, and hypothermia. The end point for each rabbit was determined after consultation between the Study Director and the veterinarian, and was based on the inability to provide clinical support for adequate relief from pain or distress due to progression of rabbitpox disease. All surviving animals were euthanized at the end of the study (Study Day 12). Euthanasia was performed by IV injection with commercial euthanasia solution (SleepAway, Lot# 471330, exp. 4/2013).

### GLP Quality Assurance Auditing

To assure that all of the mouse studies complied with GLP regulations described in 21 CFR Part 58, James Smith, PhD., RAC served as the quality assurance auditor. For all studies conducted, phase audits were completed for preparation of the monoclonal antibodies prior to dosing, the dosing itself, and monitoring of the mice by body weights and clinical scoring. Under GLP Study 1, a person not involved in dose administration, clinical observations, or clinical scoring of progressive vaccinia set up one-way blinding. Due to the nature of study 2 and study 3, one-way blinding was not set up. At the end of each study, the raw data and final report of the studies were audited and evaluated for compliance. Compliance was met in all studies.

### Antibodies

The hmAbs were previously described [36], [37]. Human anti-dinitrophenol (DNP) IgG1 mAb was derived from a Chinese hamster ovary (CHO) transfectant clone kindly provided by Hideaki Yoshida from Kyowa Hakko Kirin Co., Ltd Tokyo, Japan. For antibody purification, CHO stable transfectants were cultured in Wave™ bioreactors with serum-free medium. The purified antibodies were quantified by the Lowry protein assay using bovine IgG (Pierce Biotechnology) as a standard and they were stored in aliquots at −80°C and diluted in phosphate-buffered saline (PBS) immediately prior to injection as needed.

VIG was provided by Cangene Corporation (Winnipeg, MB, Canada). VIG was stored at 4°C and diluted in PBS immediately prior to injection, as needed. Human recommended dose was 50 mg/kg [21]. Maximum adult BALB/c SCID female mouse mass was 25 g; therefore the human equivalent VIG dose for a mouse based on mass was calculated to be 1.25 mg.

### Quantitative Real Time PCR

Viral load in rabbit blood throughout the study and in tissues at euthanasia were measured using a pan-orthopoxvirus HA gene-specific qPCR. DNA was isolated from whole blood using QIAamp DNA mini kit (Qiagen, Valencia, CA). DNA was extracted from tissues collected at euthanasia using proteinase K digestion followed by phenol/chloroform method. The assay was performed in a Taqman ABI 7900 sequence detector (Applied Biosystems, Carlsbad, CA). The PCR consisted of amplification of HA gene using pan-orthopox primers 5′-GATGATGCAACTCTATCATGTA-3′ and 5′-GTATAATTATCAAAATACAAGACGTC-3′, and probe 6FAM AGTGCTTGGTATAAGGAG MGBNFQ. DNA and standard samples were amplified in 45 cycles following the conditions: Denaturation at 95°C for 2 minutes, annealing and extension at 60°C for 20 seconds.

#### Virus neutralization assays

VeroE6 cells were seeded at 1.5×10^5^ cells/well into 24-well Costar plates (Corning Inc, Corning, NY) and used the following day (75–90% confluence). Diluted mAbs samples (10 µg/mL, final concentration) were incubated for 1 hour at 37°C, 5% CO_2_, in an equal volume (50 µL) of sonicated VACV_WR_ MV in the presence of 1% sterile baby rabbit complement (final concentration) (Cedarlane Laboratories, Ontario, Canada). VACV MV samples plus 1% baby rabbit complement were used as negative controls. Plaque assays were then done as described previously [36].

### Statistical Analyses

Tests were performed using Prism 5.0 (GraphPad, San Diego, CA, USA). The significance of the survival curves was calculated using Mantel-Cox statistical analysis of Kaplan-Meier curves. Statistical significance of cumulative weight loss was calculated as the net area under the curve (AUC; body weight versus time) for each mouse and then the statistical significance was determined between experimental groups by two-tailed unpaired Student’s *t*-tests with 95% confidence intervals without assuming a normal distribution (Welch’s correction). Statistical analyses of time to 5% weight loss was carried out by tabulating the days until weight first dropped to <95% of starting weight for each mouse, and the statistical significance was calculated using two-tailed unpaired Student’s *t*-tests with 95% confidence intervals without assuming a normal distribution (Welch’s correction). Clinical score statistical analyses from a single time point were carried out using the Mann-Whitney U test. Cumulative clinical score data was calculated as the AUC (clinical score versus time) for each mouse and the statistical significance was determined between experimental groups by two-tailed unpaired Student’s *t*-tests with 95% confidence intervals without assuming a normal distribution (Welch’s correction). Unless otherwise indicated above, statistics were carried out using two-tailed unpaired Student’s *t*-tests with 95% confidence intervals.

For rabbit studies, statistical analyses of the data were performed using GraphPad InStat.

Version 3.05 to compare peak body weight, peak body temperature and peak clinical signs scores between groups.

## Results

### *In vivo* Protective Efficacy of Pre-Exposure Treatment With Anti-Poxvirus Human Monoclonal Antibodies

**Figure 1:**
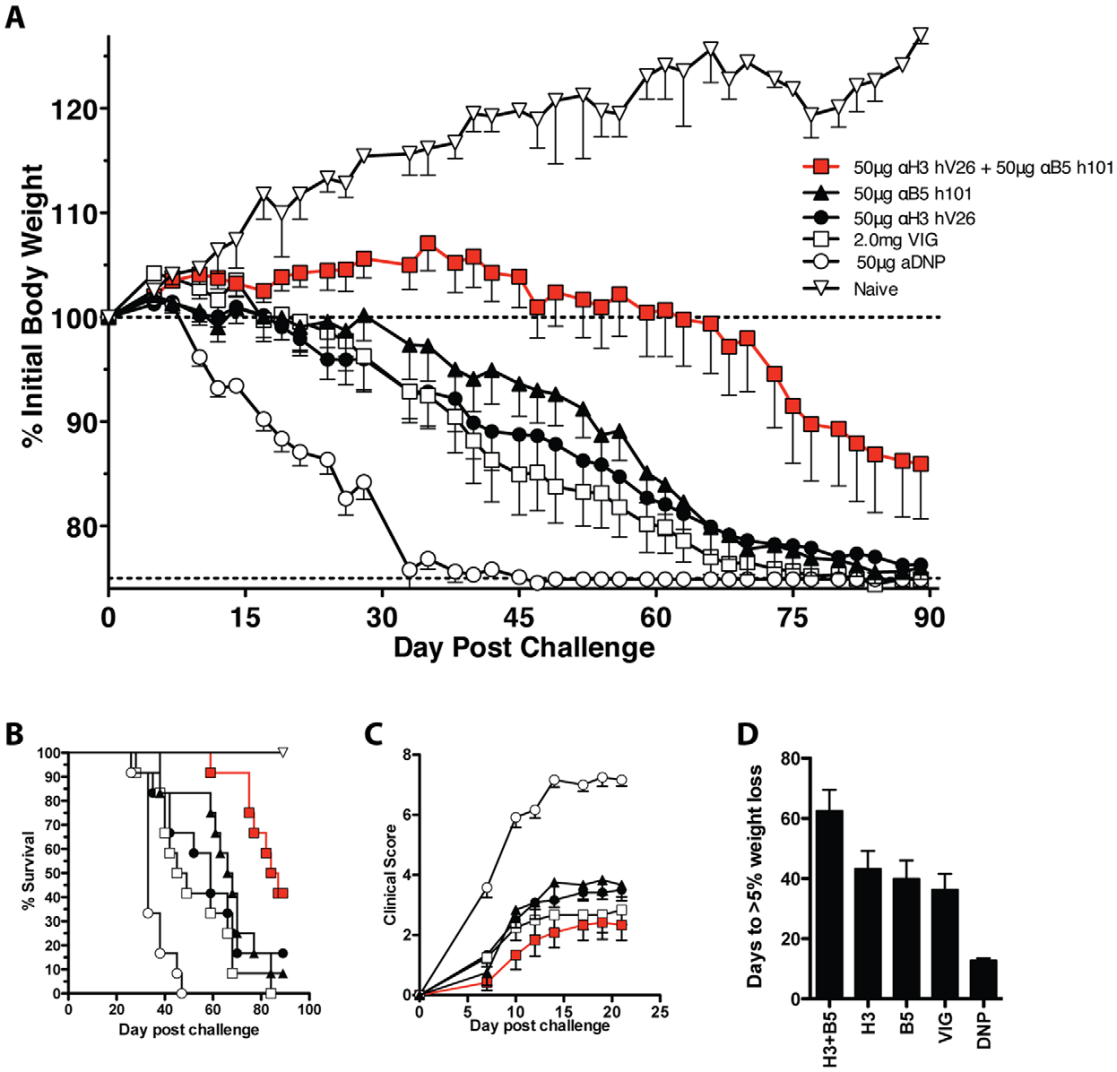
Pre-exposure hmAb protection of SCID mice. Severe combined immunodeficiency (SCID) mice were treated with a single dose of human anti-H3 hV26, anti-B5 h101, a combination of the two, 2.0 mg of vaccinia immune globulin (VIG), or negative control anti-DNP mAb at day -1 and then infected intravenously via tail vein with 5×104 PFU of vaccinia virus, ACAM2000 lab stock strain; n = 12/group, except for the naïve control [n = 2]. (A) Weights. (B) Survival. (C) Clinical scores. (D) 5% weight loss.

In previous experiments, we have shown that a combination of anti-H3 hV26 and anti-B5 h101 has significantly better protective efficacy than VIG against lethal VACV infection of SCID mice when treatments were provided before infection [36]. Those results and others [37], [38] suggested that combination hmAb treatment is a potential new anti-smallpox therapeutic. One reason to utilize an immune-compromised mouse model is to model disease of immune-compromised humans, who are contraindicated for receipt of the smallpox vaccine and are a primary vulnerable population. In response to those results, we next designed a GLP compliant (21 CFR part 58) set of studies. GLP compliant study design introduced one way blinding, an outside auditor, and numerous additional standardizations (see Methods). The first GLP animal study was designed to re-test the efficacy of anti-H3 hV26 and anti-B5 h101 separately or in combination with each other, in comparison to licensed VIG. Treatment of SCID mice with hmAbs was done prior to VACV infection. Uncontrolled VACV infection results in progressive weight loss, development of skin lesions, and death (Figure 1 and references [24], [30], [36], [39], [40]). Significant protection against weight loss was observed with the combination treatment of 50 µg of anti-H3 hV26 and 50 µg of anti-B5 h101 versus 2.0 mg of VIG (P = 0.0016, Figure 1a), and versus control hmAb DNP (P<0.0001, Figure 1a). Treatment with anti-H3 hV26 alone or anti-B5 h101 alone provided equivalent protection to that of VIG (P = NS), and significant protection against weight loss compared to control hmAb DNP was observed (P = 0.0014 and 0.0002). A significant increase in survival was observed for the combination group versus VIG (P<0.0001, Figure 1b). Anti-H3 hV26 or anti-B5 h101 alone provided survival improvement equivalent to VIG (P = NS) and significantly better than the control group (P = 0.0003 and P<0.0001, Figure 1b). The combination group exhibited significantly improved protection against overall weight loss compared to either anti-H3 hV26 alone (P = 0.0037) or anti-B5 h101 alone (P = 0.0099). Pox lesions were quantified by examining the number and severity of pox lesions developed on the tail and footpads. Clinical scores of animals provided anti-H3 hV26 and anti-B5 h101 in combination were not significantly different from VIG treated animals at day 19, a time point where lesion severity has plateaued in most animals (P = NS, Figure 1c). However, individual hmAbs alone showed significant protection compared to the DNP control group (P<0.0001 for each, Figure 1c). As a measure of early disease progression, the number of days until 5% weight loss was quantified. The combination treatment group showed significant improvement against VIG (P = 0.0071, Figure 1d), while individual mAbs did not show significant improvement over VIG (P = NS). Individual mAbs versus the control DNP group did show significant improvement (P = 0.0010 and P = 0.0004, Figure 1d). These results reaffirmed the effectiveness of combination treatment with anti-H3+B5 hmAbs pre-exposure.

A second study was designed to assess whether anti-H3+B5 hmAb treatment could ameliorate disease or cure VACV infected mice when provided post-exposure. Significant protection against weight loss was observed with the combination treatment versus the control DNP group when virus was provided intravenously through the tail vein (t.v.) for both groups (P = 0.0334). As a measure of early disease progression, the number of days until 5% weight loss was quantified. This quantification also showed significance (P = 0.0130, both groups with t.v. injection). Survival was also significantly improved with the combination treatment versus the control group (P = 0.0265, both groups with t.v. injection). Clinical scores were taken at day 25, and hmAb anti-H3+B5 treated mice exhibited significant protection against lesions (P = 0.0375, both groups with t.v. injection).

**Figure 2:**
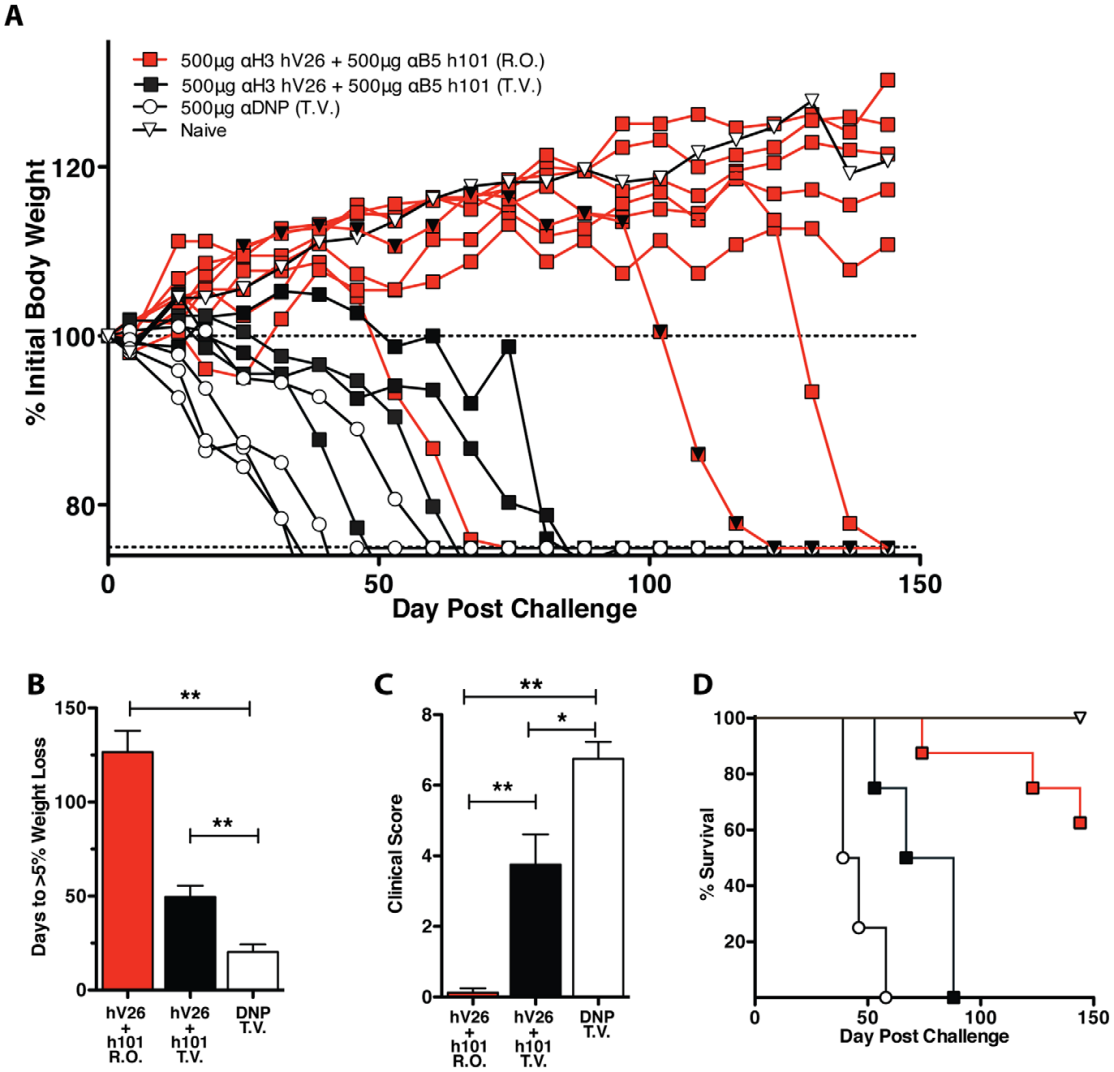
Post-exposure hmAb protection of SCID mice. (**A**) SCID mice were treated with a 500 µg dose of human anti-H3 hV26 and anti-B5 h101 in a split dose of 200 µL retro-orbital and 200 µL intraperitoneal on day 1 post-infection. A booster dose of 500 µg of combination therapy was provided day 14 intraperitoneal. All mice were infected with 5×104 PFU of vaccinia virus, ACAM2000. Half of the mice in the combination group were given virus intravenously through the tail vein T.V. (n = 8), while the other half received virus intravenously R.O. (n = 8). All anti-DNP control mice received virus T.V. (n = 4). (**B**) Days to 5% weight loss. (**C**) Day 25 clinical scores. (**D**) Survival.

In previous studies, all virus infections were via lateral tail vein injection (t.v.). In this study, a group with retro-orbital intravenous injection (r.o.) was added. Retro-orbital injections are more consistent, resulting in less mouse-to-mouse variability. Outstanding protection against weight loss was seen was for the hmAb anti-H3+B5 treated mice with r.o. infection versus the control group given virus t.v. (P<0.0001; Figure 2a). Unexpectedly, the protection against weight loss was significantly better for the combination treatment when virus was provided r.o. versus t.v. (P = 0.0002; Figure 2a). A significant increase in survival was also observed for the hmAb anti-H3+B5 treated mice given virus r.o. versus t.v. (P = 0.0034, Figure 2d). Clinical scores were significantly better in anti-H3+B5 treated mice given virus r.o. versus t.v. (P = 0.0246; Figure 2c), as was days to 5% weight loss (P = 0.0002; Figure 2b). Overall, the hmAb anti-H3+B5 treated mice infected r.o. appeared to show consistent long term protection against VACV infection. However, this experiment lacked the appropriate r.o. control group, which precipitated the follow-up study, in which all groups received virus through the retro-orbital route.

**Figure 3:**
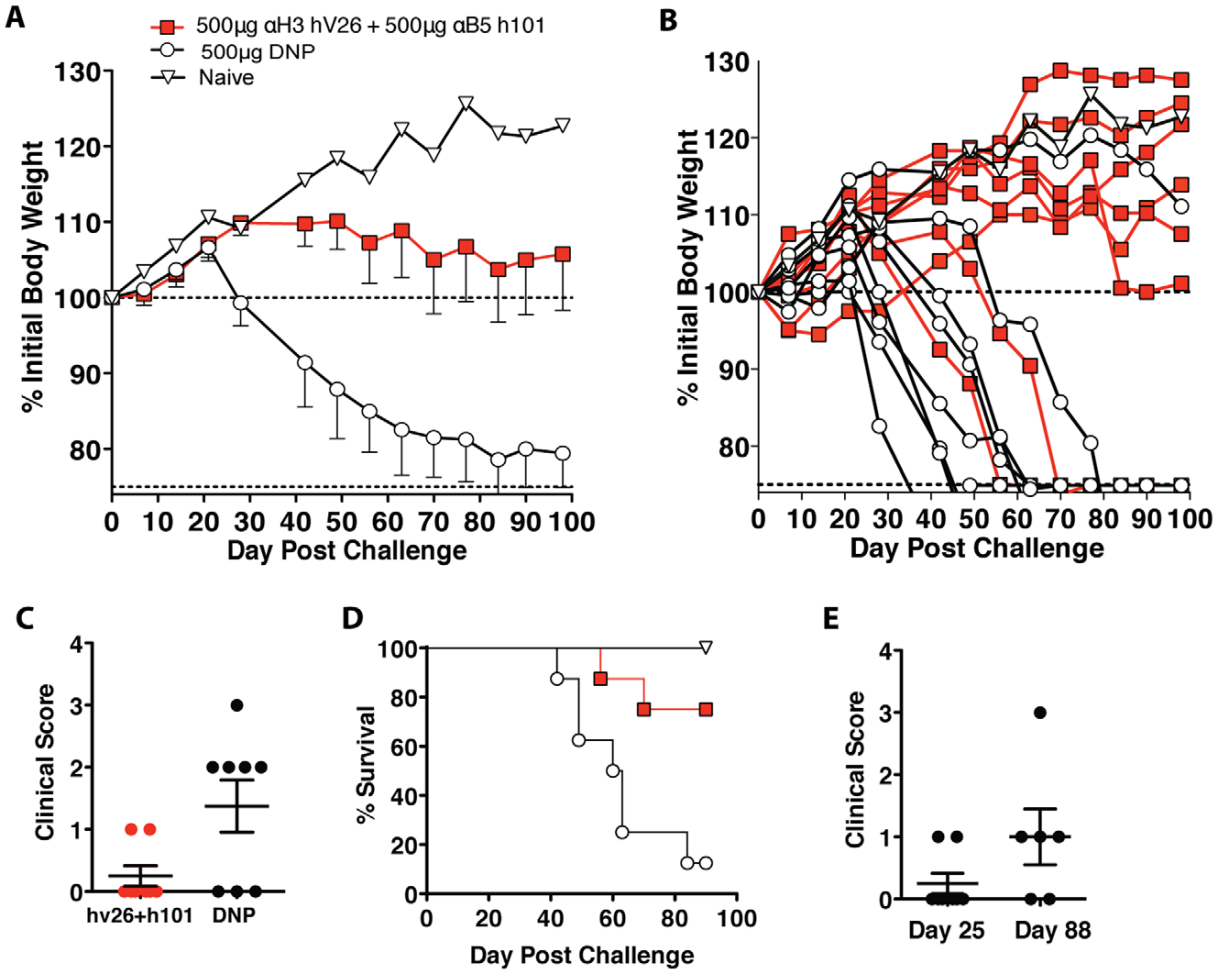
Post-exposure hmAb protection with single animal housing. (**A**) SCID mice were treated with a 500 µg dose of human anti-H3 hV26 and anti-B5 h101 retro-orbitally on day 1 post-infection. A booster dose of 500 µg of the combination therapy was provided to the mice on day 11 intraperitoneal. All mice were infected with 5×104 PFU of vaccinia virus, ACAM2000 retro-orbitally on day 0. For both groups n = 12. 4 mice in each group were sacrificed at day 28 for other analyses not shown. (**B**) Individual mice weights. (**C**) Clinical scores day 25. (**D**) Survival. (**E**) Clinical scores of the combination anti-H3+B5 group on day 25 versus day 88.

In the third study, again done under GLP conditions, we additionally aimed to assess the efficacy of the combination therapy post-exposure while utilizing single mouse housing. The rationale for single mouse housing in this experiment was to minimize the possibility of mouse-to-mouse spread of VACV. In the previous study it appeared that some of the animals were protected for a significant period of time, but they were co-housed with animals that succumbed to infection, developed extensive pox lesions, and required euthanasia when their body weight dropped below 75%. Pox lesions have high concentrations of virus in the scabs, which may provide a continuous source of virus within a cage, resulting in re-infection of co-housed animals. Single mouse cage housing avoids this problem. The experiment was also designed so that all mice were infected with VACV via the r.o. route. Anti-H3+B5 hmAbs were provided at day +1 and day +14 post-infection. Significant protection against weight loss was observed with the anti-H3+B5 hmAb group versus the control group (P = 0.0056; Figure 3a–b). Clinical scores at day 25 showed significant improvement in mice provided anti-H3+B5 hmAbs (P = 0.0341; Figure 3c). Survival was also significantly improved in the anti-H3+B5 hmAb group (P = 0.011; Figure 3d). At the termination of the study, day 98, six out of eight mice in the anti-H3+B5 hmAb treated group survived, whereas only 1 mouse out of eight survived in the control group. Of the six mice in the anti-H3+B5 hmAb group alive at day 98, all of the mice were above 100% of their pre-study body weight. An additional clinical score was measured at day 88 to evaluate any long-term changes in clinical status. In comparing clinical score data for the combination group on day 25 versus day 88 showed no clinical significance (P = NS; Figure 3e). Four of the eight mice in exhibited no disease progression (weight > 100% and no clinical score change after day 25.) Two additional mice showed minor disease progression and some clinical score change. These results show that post-exposure treatment with anti-H3+B5 hmAbs could potentially cure animals of a lethal poxvirus infection.

### *In vivo* Protective Efficacy of Post-Exposure Treatment With Anti-Poxvirus Human Monoclonal Antibodies in Lethal Rabbitpox Challenge

**Figure 4:**
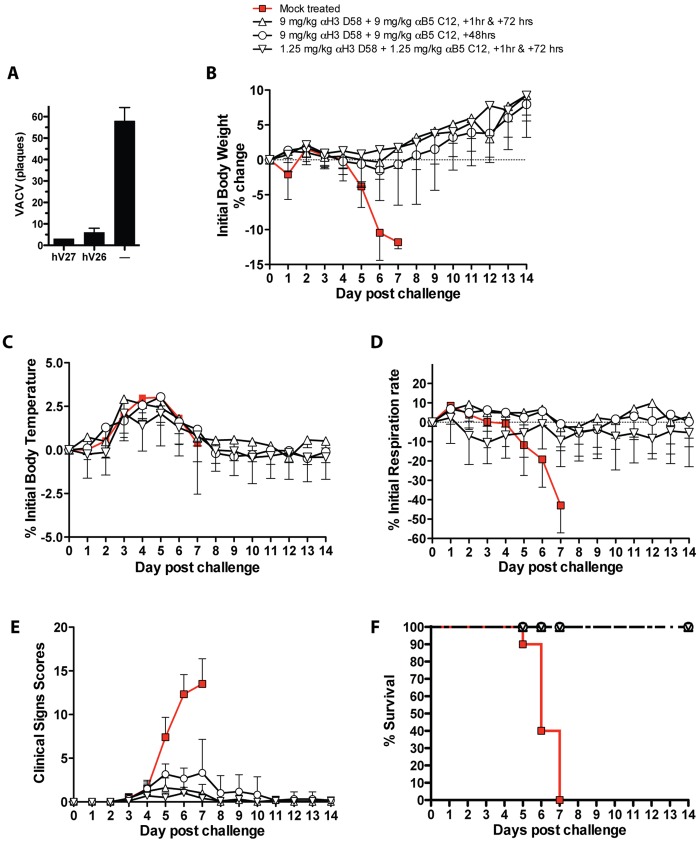
Post-exposure anti-H3+B5 hmAb protection in rabbits. (**A**) VACV neutralization in vitro. (**B–F**) Post-exposure protection of rabbits against rabbitpox. 32 New Zealand White (NZW) rabbits were challenged with 1x105 PFU of RPXV intranasally. Group 1 (n = 10) received no treatment, Group 2 (n = 6) and group 4 (n = 10) received two I.V. injections of antibody cocktail at 9 mg/kg and 1.25 mg/kg respectively, one hour and 72 hours post-challenge. Group 3 (n = 6) received a single I.V. injection of antibody cocktail at 9 mg/kg 2 days post-challenge. (**B**) Body Weights. (**C**) Percent change from initial body temperature. (**D**) Percent change from initial respiration rate. (**E**) Clinical scores. (**F**) Percent survival.

**Figure 5:**
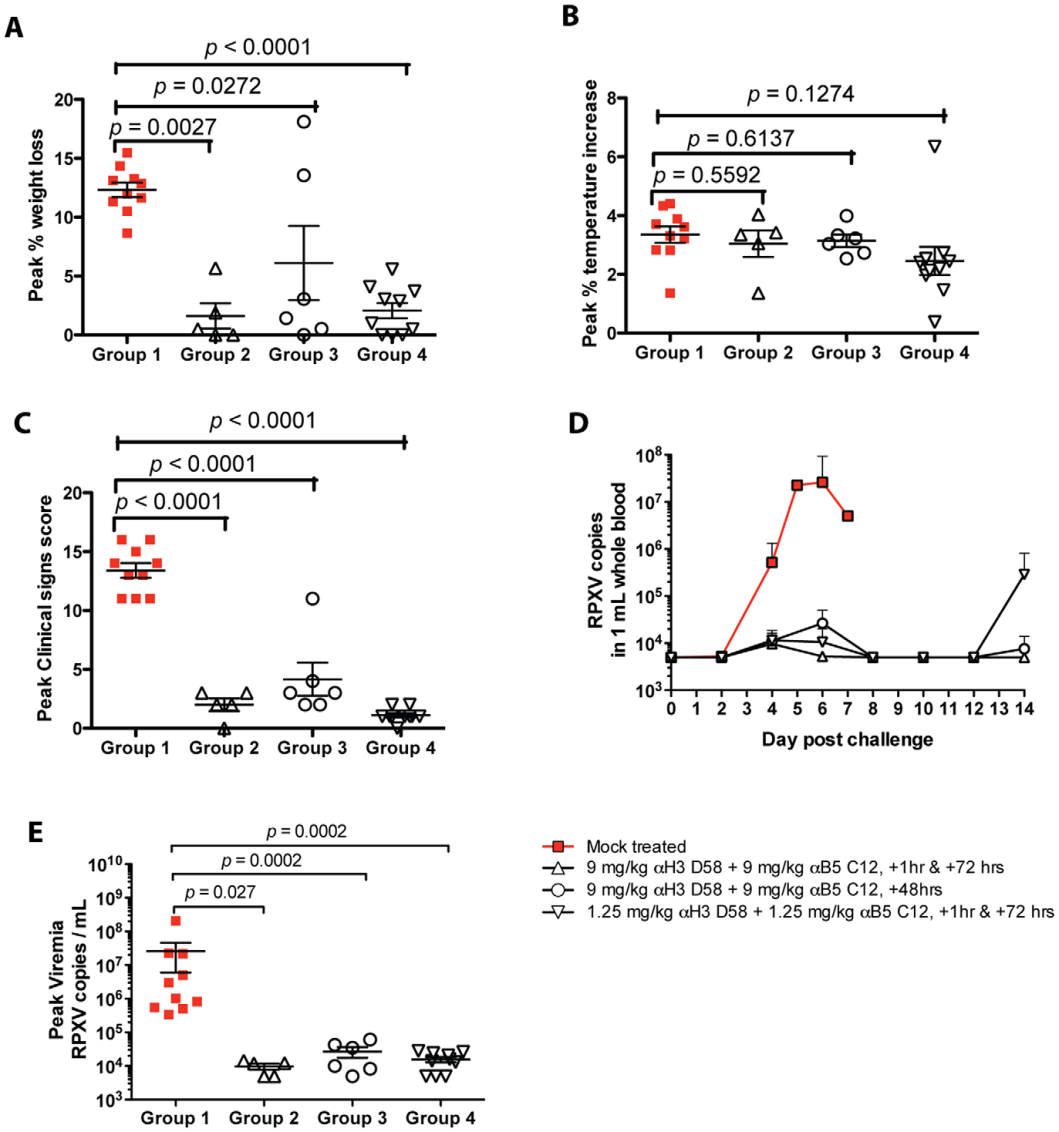
Post-exposure anti-H3+B5 hmAb protection in rabbits. (**A**) Peak percent weight loss amongst groups. (**B**) Peak percent temperature increase. (**C**) Peak clinical score. (**D**) RPXV copies in 1 mL of whole blood. (**E**) Viremia at peak disease (RPXV copies/mL).

Having completed a series of studies in immunocompetent and immunocompromised mice (Fig. 1, 2, 3 and refs. [36], [37], [38]), we then progressed to studies of the efficacy of the hmAbs in larger animal. Rabbitpox (RPXV) is a highly virulent orthopox infection of rabbits with clinical symptoms similar to that of smallpox in humans. We therefore examined whether the hmAbs could protect rabbits against death from RBPX, when the hmAbs were provided as post-exposure prophylaxis. Four groups of rabbits were exposed to a lethal dose of RPXV via intranasal route on Day 0; then treated via intravenous injection with a combination of hmAbs to H3 and B5 antigens. A new anti-H3 hmAb subclone (hV27) was used that exhibited no cross reactivity in human tissue cross reactivity studies (data not shown). HmAb hV27 differs from hmAb hV26 by only 4 amino acids. HmAb hV27 has equivalent or better VACV *in vitro* neutralization activity (Figure 4a) and *in vivo* protection of SCID mice compared to hV26 (data not shown). Group 2 (n = 6) and Group 4 (n = 10) received treatment twice on Day 0 at 1 hr and Day 3 post challenge at doses of 9 mg/kg and 1.25 mg/kg, respectively. Group 3 (n = 6) received a single treatment of 9 mg/kg at Day 2 post challenge. Group 1 (n = 10) did not receive the treatment and served as challenge control group. All rabbits were monitored for clinical signs of pox disease for 14 days. One rabbit in Group 2 broke a leg on Day 0 and was euthanized to relieve pain and distress and was thus excluded from analysis. As expected, all 10 rabbits in the untreated challenged group (Group 1) succumbed within 5 to 7 days following challenge with RPXV after they developed severe pox disease that warranted euthanasia. All animals treated with two high doses of the antibody cocktail at 1 hr and 72 hrs post challenge survived RPXV infection until the end of the study Day 14 (Figure 4f). All animals treated with two low doses of the antibody cocktail at 1 hr and 72 hrs post challenge survived RPXV infection (Figure 4f). Furthermore, all animals treated with a single dose of the antibody cocktail at 48 hrs post challenge survived RPXV infection (Figure 4f). Following challenge, the rabbits in control Group 1 experienced increase in body temperature, which peaked between Days 4 and 5. Increase of body temperature starting at Days 1 or 2 post-challenge was detected in all treated animals in Groups 2–4, which returned to normal ranges by Day 7 to 8 post challenge in most cases (Figure 4c, 5b).

Daily body weight of all animal groups was monitored following challenge. As expected, all animals in the challenge control group (Group 1) experienced a steady weight loss, which continued until they were euthanized by Days 5–7, when the weight loss reached a range of 10.5% to 15.5% (Figure 4b, Figure 5a). In Group 2 all animals continued to gain weight following challenge, with the exception of two animals that experienced 5.7% and 1.9% weight loss by Day 5 post challenge, respectively. However, these two rabbits quickly regained normal weights by Day 7 post-challenge. Overall, significant protection against weight loss was observed for all treatment groups in comparison to the untreated group (Group 4 vs. 1, P<0.0001. Group 3 vs. 1, P = 0.0272. Group 2 vs. 1, P = 0.0027. Figure 4b, 5a).

Respiration rate (i.e. the number of breaths per minute) was recorded daily for all animal groups. All rabbits in Group 1 experienced typical respiratory distress due to RPXV infection, evidenced by a slight increase in respiration rate within the first 2 to 3 days after challenge and then a sustained decrease thereafter of increasing severity until euthanasia. Group 1 rabbits experienced severe respiratory distress with difficulty breathing, open mouth breathing and severe lung sounds warranting euthanasia. All treatment regimens administered to Groups 2–4 prevented the decrease in respiration rate shown in the challenge control group (Figure 4d).

Following challenge, clinical observations were recorded daily for all the rabbits. Severity of clinical signs of pox disease progression including depression, weakness, dehydration, dyspnea, cough, loss of appetite, nasal and ocular discharge, and edema, was given a score of 0, 1, 2 or 3 corresponding to no sign, mild, moderate or severe levels, respectively. As expected, by Day 3 to 4, the Group 1 control animals began showing clinical signs of disease of mild to severe loss of appetite. By Day 4 post-challenge, the rabbits began experiencing nasal and ocular discharges, which worsened until euthanasia. By Day 5 post-challenge, all the rabbits experienced mild to severe edema within the face. By Day 5 post-challenge, the majority of Group 1 rabbits began experiencing mild to moderate dehydration and depression, which worsened. The overall clinical signs score of Group 1 animals reached between 11 and 16 by euthanasia day. Overall, clinical scores showed statistically significant differences between all treated groups and the untreated group (P<0.0001, Figure 4e, 5c).

**Figure 6:**
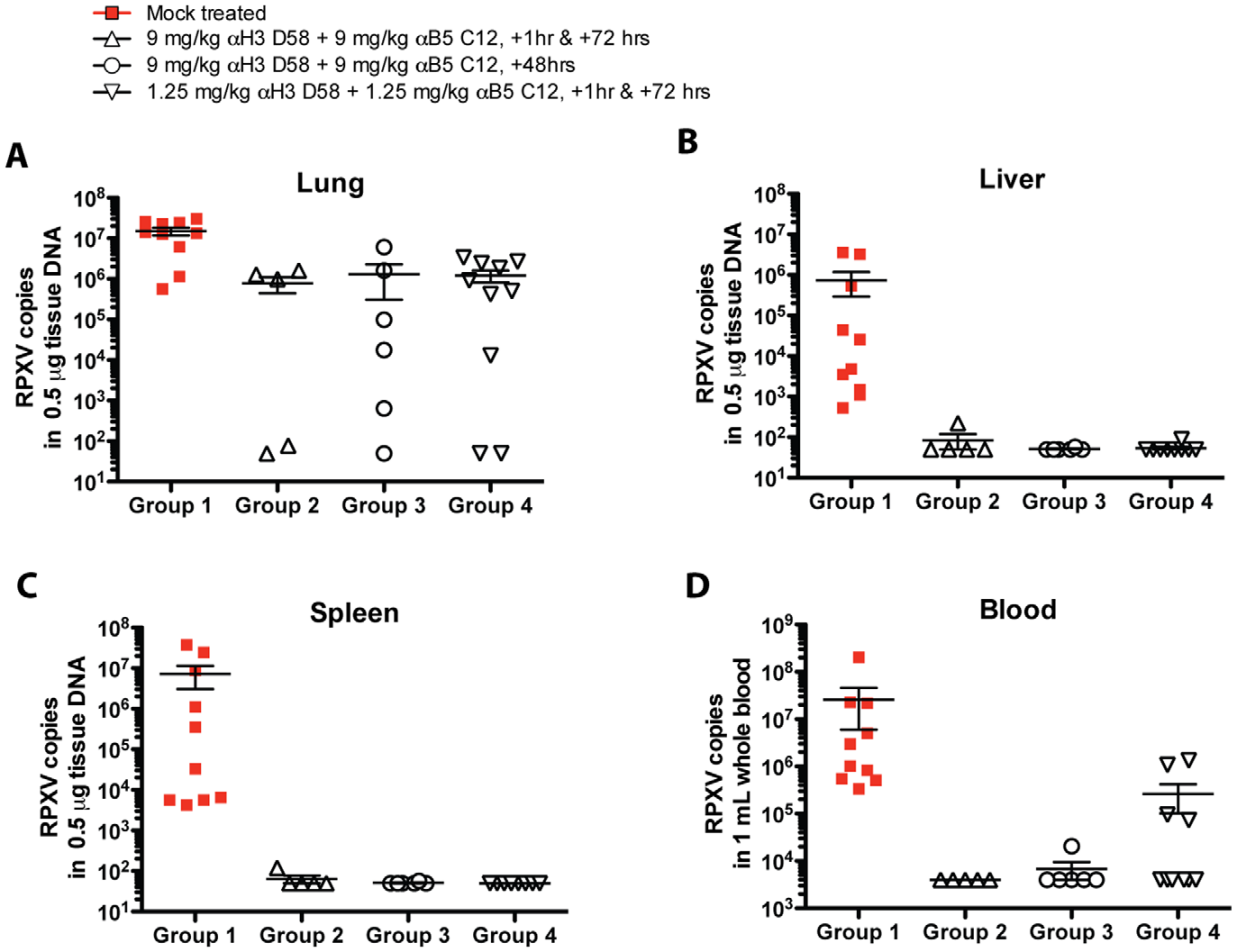
RPXV in Lung, Liver, Spleen, and Blood. RPXV in the liver, spleen, and blood of infected NWZ rabbits.

Viral load kinetics were measured in the blood throughout the study by quantitative real time PCR. As expected, significant viremia was detected in all Group 1 rabbits by Day 4 post-challenge. The viremia continued increasing to reach ranges between 10^6^–10^8^ by Days 5–7 (Figure 5e–f). Similarly, higher tissue viral loads were detected in lung, liver, and spleen collected from the Group 1 control animals at euthanasia, indicative of a broad viral RPXV dissemination (Figure 6). In contrast, in Group 2 rabbits that received 9 mg/kg hmAbs twice post-challenge, a low level viremia was only measured in three rabbits on Day 4 post-challenge, which decreased to below the detection limit by Day 6–8. Viremia was below the detection limit in the 2 other rabbits throughout the study duration. In Group 3 rabbits that received a single dose of hmAbs at 48 hrs post-challenge, a low peak viremia was detected at Day 6, which returned to undetectable levels by Day 8 post-challenge. Viremia was below the limit of detection for one rabbit in Group 3. In Group 4, rabbits that received 1.25 mg/kg hmAbs, a low level viremia was detected at Day 4 and declined by Day 6. Viremia was below the limit of detection for 3 animals in Group 4 throughout the course of the study. Overall, there was a statistically significant difference of peak viremia between control Group 1 and treatment Groups 2–4 (each P = 0.0001). Interestingly, a rebound of viral load was detected on Day 14 in the blood of three rabbits in Group 4. While viremia was well controlled in all treated rabbits, virus was present in the lung harvested on Day 14 post-challenge regardless of treatment regimen. However, virus replication in liver and spleen was controlled to undetectable levels in all treatment groups (P<0.001, Figure 6).

**Figure 7:**
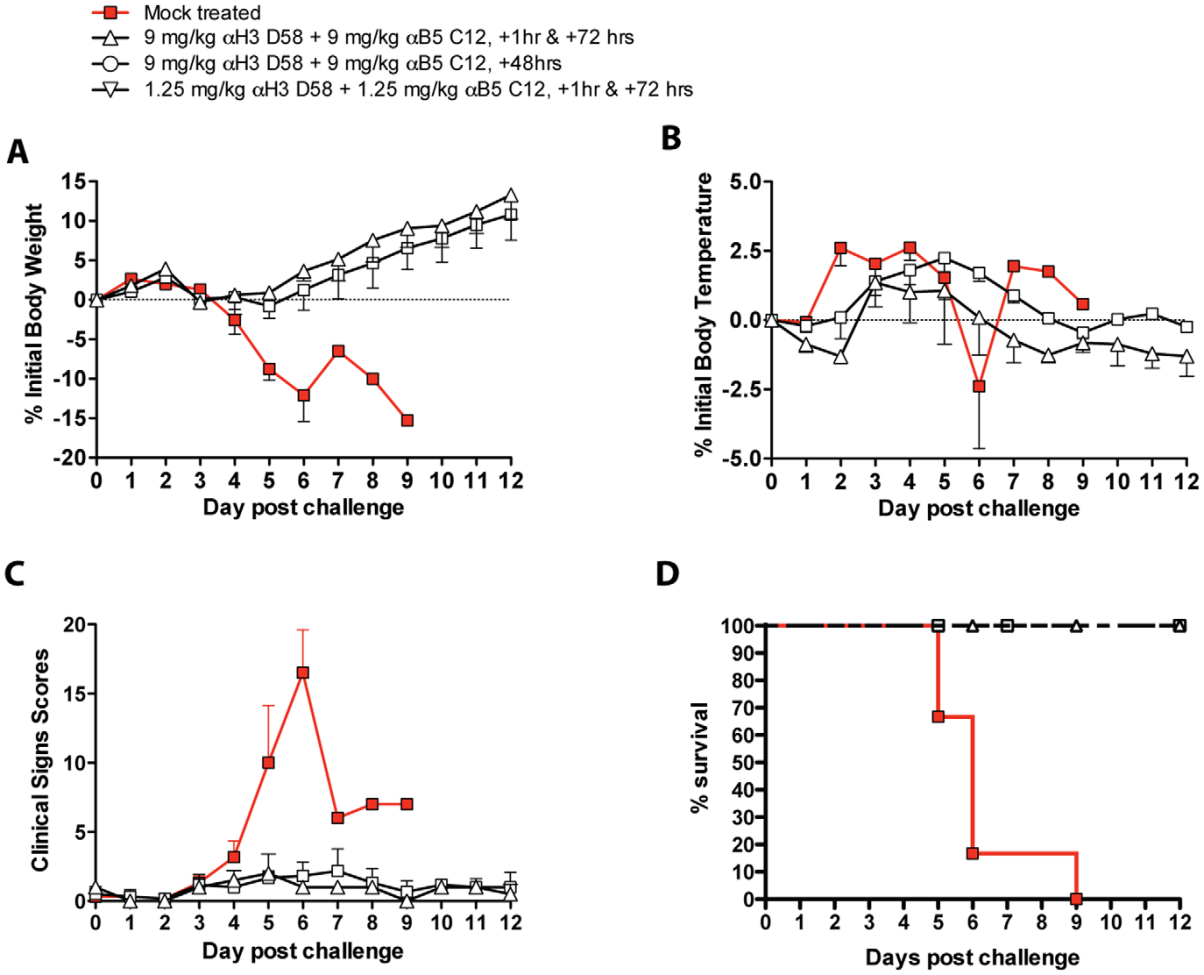
Post-exposure protection in rabbits with single hmAb. (A) Body Weights. 14 New Zealand White (NZW) rabbits were challenged with 1×105 PFU of RPXV intranasally. Group 1 (n = 6) received no treatment, Group 2 (n = 6) received two I.V. injections of anti-B5 h101 alone at 9 mg/kg, one hour and 72 hours post-challenge. Group 3 (n = 2) received two I.V. injections with the antibody cocktail at 9 mg/kg, one hour and 72 hours post-challenge. (**B**) Percent change in initial body temperature. (**C**) Clinical scores. (**D**) Percent survival.

**Figure 8:**
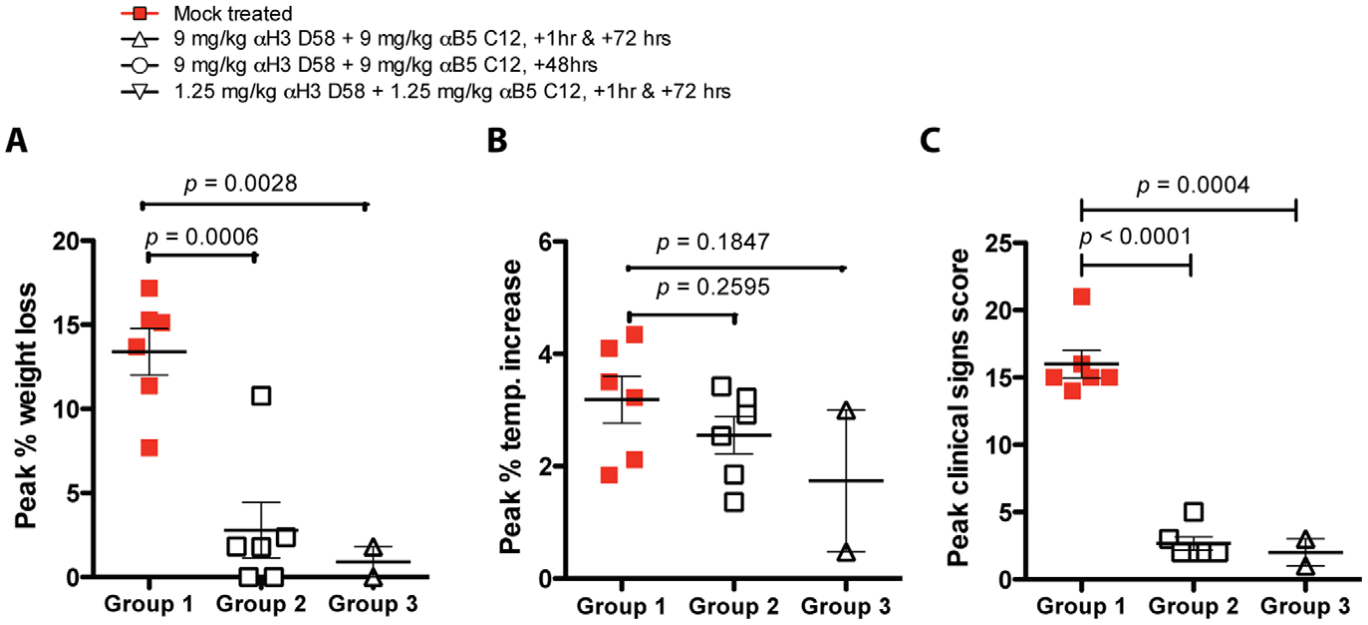
Post-exposure protection in rabbits with single hmAb. (**A**) Peak percent weight loss amongst groups. (**B**) Peak percent temperature increase. (**C**) Peak clinical score.

Given the strong success of the first rabbit study, a second study was designed to determine whether single hmAb treatment post-exposure would suffice to protect against a lethal RPXV challenge. A total of 14 rabbits distributed into 3 groups were challenged intranasally with 1×10^5^ PFU of RPXV. Group 1 (n = 6) did not receive treatment and served as the study negative control. Group 2 (n = 6) received two intravenous injections of antibody h101 alone at 9 mg/kg, 1 hour and 72 hours following challenge. Groups 3 (n = 2) received two intravenous injections with the antibody cocktail at 9 mg/kg, 1 hour and 72 hours post-challenge. Daily body weight was recorded following RPXV challenge for all rabbits. Rabbits were euthanized if they reached greater than 15% of pre-challenge body weight. As expected, rabbits in Group 1 that did not receive treatment began losing weight starting at Day 2 to 5 post-challenge and continued to lose weight until they were euthanized (Figure 7a). In contrast, two rabbits in Group 2 that received hmAb h101 alone did not experience any weight loss. Three Group 2 rabbits experienced slight weight loss (2.38%, 1.83%, 1.79%). One rabbit did experience significant weight loss that reached 10.78% at Day 6 post-challenge before improving over subsequent days. However, this animal only exhibited minor clinical signs of disease. Rabbits in Group 3 that received hmAbs combination treatment did not lose significant weight (Figure 7a). Statistical analysis of mean peak weight loss between groups revealed that the difference in weight loss between Group 1 and Group 2 and Group 1 and 3 was statistically significant (P = 0.0006 and P = 0.0028 respectively, Figure 8a). Moreover, there was no statistical difference in mean peak weight loss between Group 2 that received h101 alone and Group 3 that received mAbs combination h101 and hV27 (P = 0.5566).

Body temperature was monitored daily for all rabbits. Rabbits in Group 1 that did not receive treatment experienced typical body temperature spikes post challenge. Although rabbits in Group 2 that received h101 hmAb treatment alone experienced increase in body temperature, it was to a lesser extent than animals in Group 1 (Figure 7b). Similarly, rabbits in Group 3 that received anti-H3+B5 hmAbs experienced temperature spikes (Figure 7b). Statistical analysis of mean peak percent temperature increase between groups revealed that the difference in body temperature percent increase between Group 1 and Group 2 and Group 1 and Group 3 were not significant (P = 0.2595 and P = 0.1847 respectively; Figure 8b).

Rabbits were monitored daily for clinical signs of pox disease and behavioral changes. As expected, rabbits in Group 1 that did not receive any treatment began developing signs of disease progression starting at Day 3 post-challenge and continued to worsen until they were euthanized due to severe pox disease. The animals began experiencing mild loss of appetite by Day 1 post challenge, which translated to weight loss by Day 3–4. By Day 4 post challenge, rabbits began experiencing signs of dyspnea as evidenced by difficulty breathing and drop in respiration rate. On Day 5 post challenge, rabbit #682 exhibited signs of severe respiratory distress as evidenced by open mouth breathing and cyanotic stage, which warranted euthanasia. Rabbit #683 experienced severe respiratory distress with open mouth breathing and lung sounds was also euthanized on Day 5 post-challenge. On Day 6 post-challenge, rabbit #680 was euthanized due to severe lethargy, depression and dehydration accompanied by severe ocular and nasal discharges. By Day 6 post-challenge, rabbit #691 exhibited severe depression and weakness and severe ocular discharge coupled with moderate dyspnea warranting euthanasia. Rabbit #694 also developed severe respiratory distress and depression by Day 6 post-challenge, which warranted euthanasia. Rabbit # 692 demonstrated moderate depression, lethargy and dyspnea coupled with moderate ocular and nasal discharges was euthanized on Day 9 post-challenge due to severe loss of appetite, which translated to greater than 15% weight loss. Interestingly, rabbits in Group 2 that received hmAb h101 treatment did not develop any substantial clinical disease signs following RPXV challenge (Figure 7c, Figure 8c). All rabbits in this group did not develop any nasal or ocular discharges except for one that exhibited mild ocular discharge. Three rabbits developed mild dyspnea while the remaining rabbits in this group did not develop any sign of respiratory distress at any time during the challenge phase. Only one rabbit exhibited severe loss of appetite on Day 7 post-challenge, which lasted only 1 day. Similarly, rabbits in Group 3 that received hmAbs combination of h101 and hV27, did not develop any sign of respiratory distress, depression, weakness or discharges (Figure 7c, Figure 8c). Statistical analysis of mean maximum clinical signs cumulative score between groups revealed that the difference between Group 1 and Group 2 was very significant, as well as between Group 1 and 3 (P<0.0001 and P = 0.0004 respectively; Figure 8c). There was, however, no statistical difference between Group 2 that received anti-B5 alone and Group 3 that received anti-H3+B5 hmAbs (P = 0.5370). Overall, single treatment with hmAb h101 was sufficient to protect rabbits from death and disease when provided post-exposure.

## Discussion

Antibodies against VACV and smallpox are extremely important components of the smallpox vaccine-mediated protection [27], [28]. Studies in nonhuman primates have shown that smallpox vaccine elicited antibodies are sufficient to protect macaques from lethal monkeypox infection [26], which is the closest available animal model to human smallpox. Those results are consistent with old clinical studies in humans that smallpox vaccine elicited antibodies are effective post-exposure propylaxis against variola infection. MAbs against VACV MV and EV virion forms have been previously demonstrated to be protective in a variety of small animal experimental models [32], [34], [36], [37], [38], [39], [41]. Antibodies against virulence factors can also have efficacy [42]. Our goal has been to develop human mAbs directed against known neutralizing antibody targets as therapeutics for use against smallpox or monekpox infection of humans.

Three major goals were accomplished in the studies reported here. First, mouse protection results were shown to be reproducible under GLP conditions. Second, the post-exposure prophylaxis experiments show evidence that the hmAbs may cure a lethal poxvirus infection in immunodeficient mice. This is of substantial interest for potential treatment of immunodeficient or immunocompromised humans, and warrants further investigation. Finally, two extensive rabbit studies show that hmAbs can provide robust protection against a lethal RPXV infection when provided post-exposure. These studies also show that a relatively low dose of hmAbs is required for effective protection. Overall, our studies show that our combination therapy is a promising approach as a poxvirus therapeutic for use in humans.

Development of therapeutics against potential bioterrorism pathogens (variola) or diseases for which infection in the USA is currently very rare (monkeypox) is challenging for several reasons, including the inability to directly test for efficacy in humans. There are high quality small molecule drugs being considered as treatments for smallpox infection of humans [40], [43], [44]. Our approach has been to develop human mAbs as a conservative approach to this problem, since we know that antibodies protect humans against smallpox after vaccination and hmAbs are extremely safe. This study demonstrates that hmAbs against smallpox can indeed be highly efficacious in immunocompromised mice and rabbits, warranting further studies in non-human primates.

HmAbs against variola have several potential uses in humans. The most direct use planned is treated of individuals who have been exposed to variola in a bioterrorism incident. In such a case, the hmAbs need to be effective as post-exposure prophylaxis, and most likely need to be effective at preventing smallpox when given 2–3 days after the initial virus exposure. Here we demonstrate that hV27+h101 are effective in an animal model of this situation: rabbits challenged with a lethal aerosol dose of RPXV and then provided a single dose of hmAbs two days later. All animals were protected under those conditions. Individuals who are immunocompromised are particularly vulnerable, because vaccination with conventional smallpox vaccine (ACAM2000) is contraindicated in those individuals. As such, it is important to be able to protect those individuals with therapeutics. Here we demonstrate that hmAbs are effective in an animal model of this situation: severely immunodeficient mice challenged with a lethal dose of VACV evidenced a high degree of protection when provided anti-H3+B5 hmAbs. It is also feasible that hmAbs against variola could find clinical utility in treatment of monkeypox, given that smallpox vaccine elicited antibodies are protective against lethal monkeypox infection.
